# Health 2050: The Realization of Personalized Medicine through Crowdsourcing, the Quantified Self, and the Participatory Biocitizen

**DOI:** 10.3390/jpm2030093

**Published:** 2012-09-12

**Authors:** Melanie Swan

**Affiliations:** MS Futures Group, P.O. Box 61258, Palo Alto, CA 94306, USA; E-Mail: m@melanieswan.com; Tel.: +1-650-681-9482; Fax: +1-504-910-3803

**Keywords:** personalized medicine, preventive medicine, crowdsourcing, participatory medicine, participant-centric initiatives, digital health, health empowerment, health trust communities, quantified self, future of medicine

## Abstract

The concepts of health and health care are moving towards the notion of personalized preventive health maintenance and away from an exclusive focus on the cure of disease. This is against the backdrop of contemporary public health challenges that include increasing costs, worsening outcomes, ‘diabesity’ epidemics, and anticipated physician shortages. Personalized preventive medicine could be critical to solving public health challenges at their causal root. This paper sets forth a vision and plan for the realization of preventive medicine by 2050 and examines efforts already underway such as participatory health initiatives, the era of big health data, and qualitative shifts in mindset.

## 1. Introduction

### 1.1. Contemporary Public Health Challenges

When considering the critical health challenges of the current era, it is easy to think of the 18% of the U.S. GDP being spent on health care ($8,402 per person per year in 2010) [1], health outcomes that lag those of other Organization for Economic Co-operation and Development (OECD) countries [2], the obesity epidemic (the U.S. Centers for Disease Control (CDC) estimates that 42% of American adults will be obese by 2030 as compared to 34% today) [3], aging worldwide populations [4], anticipated physician and nursing shortages, the high cost of bringing a new drug to market ($1.3 billion) [5], and the fact that 62% of bankruptcies in 2007 were medically-related [6]. In spite of these factors, this paper instead argues that the key public health challenge at present is the realization of preventive medicine. Resolving this central higher-order challenge could more expediently address the other issues which may be more symptomatic than causal. 

### 1.2. Health 2050

Health 2050 is a term used in this paper to collect attention around the idea of a practical project with a feasible time frame for a meaningful shift to preventive medicine in both individual mindset and societal institutions. Health 2050: Preventive Medicine is a meme that could expand into a conference, research institution, think tank, government initiative, or other program. The core principles involve the empowerment of the individual, at any age, to self-monitor and self-manage health and wellness, and conditions of higher risk and existing diagnosis, and further, to start doing this today with tools that are already available. 

### 1.3. Preventive Medicine

The ‘realization of preventive medicine’ is an umbrella term which subsumes personalized medicine, and also the participatory and predictive aspects of the notion of ‘4P medicine’ initially promulgated by systems biologist Lee Hood (e.g., medicine that is predictive, personalized, preventive, and participatory) [7]. This general area of personalized preventive medicine can be distinguished as a form of medicine that uses information about an individual’s genome, current biophysical measures, and environment to prevent, diagnose, and treat disease [8]. The objective of preventive medicine is also relevant as it is a much-used term with different meanings. For example, to current medical practitioners, preventive medicine may typically mean reducing hospital readmit rates for already-diagnosed and treated patients since this is where a large portion of medical costs accrue. A more general objective of preventive medicine that is more broadly applicable is to extend healthy lifespan and reduce disability. This general objective is parlayed into a working definition in this paper to discuss one aspect that can be applied most readily now by laypersons and medical practitioners, the idea of keeping populations healthy and preventing conditions from arising in the first place, especially now that data are starting to be available to identify and manage risks with greater granularity ahead of time. 

The scope and organization of this paper is to first discuss the expanded concept of health and health care that is at the heart of Health 2050: Preventive Medicine, then to look at the different dimensions of a condition’s life cycle before it becomes clinical, and finally to propose how preventive medicine may be realized through participatory health initiatives, the era of big health data, and philosophical shifts in mindset.

## 2. Health 2050: Preventive Medicine

**Figure 1:**
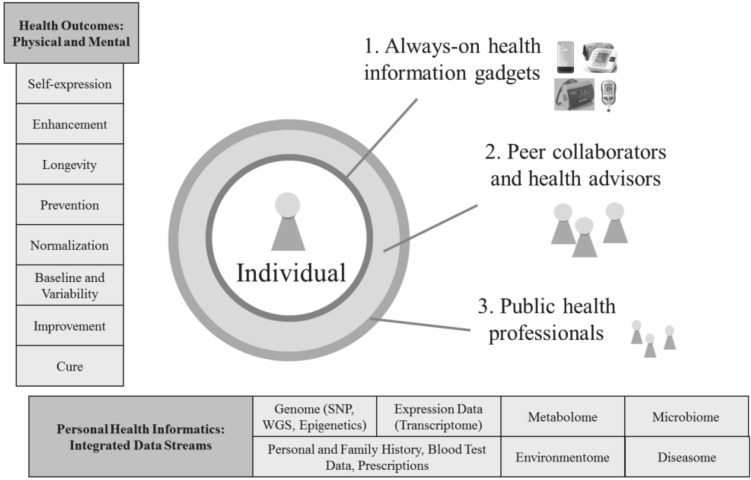
Health 2050: An expanded concept of health and health care. SNP: single nucleotide polymorphism, WGS: whole human genome sequencing.

Part of Health 2050: Preventive Medicine is realizing that there is now a much broader concept of health and health care which has not yet been fully articulated in the public dialogue. One of the crucial conceptual shifts in preventive medicine is that not just is a patient’s treatment in a personalized *n* = 1 manner, but the patient, really a participant, or simply a person, becomes the nexus of action-taking and empowerment. *N* = 1 means that the individual, now through quantified self-tracking and other low-cost newly-available tools, has the ability to understand his or her own patterns and baseline measures, and obtain early warnings as to when there is variance and what to do about this. Figure 1 depicts the expanded concept of health and health care. 

### 2.1. Greatly Expanded Range of Health Outcomes

A range of health outcomes and objectives are collected in the box on the left in Figure 1. They are greatly expanded from the traditional nearly exclusive focus on cure to branch upwards to a wider set of endpoints such as establishing baseline and variability levels of phenotypic measures in individuals, improving, normalizing, and preventing conditions, and proactively targeting longevity, enhancement, and health as a self-expression mechanism of the individual. This stratification in health outcomes is already starting to be seen in the area of physical health with new focal points on condition prevention and wellness maintenance. A similarly large, and relatively untapped area is mental health. More nascent in its problematization as a public health concern, mental health and mental performance optimization may become increasingly important as an outcome in the near future, especially with the tools of modern technology such as the intimate therapeutic delivery platform of the mobile phone. 

### 2.2. Data Stream Integration Needed for Personal Health Informatics

In the box at the bottom are examples of the different types of data that are starting to be synthesized into an overall personal health informatics picture. These include genomic data in the form of single nucleotide polymorphisms (SNPs)—locations in the genome where an individual may have a different genetic sequence than the ‘normal’ type; whole human or exome sequencing—more detailed genomic information regarding protein-coding, regulatory, and other regions of the genome; and epigenetics—genetic changes that occur during an organism’s lifetime. 

Another health data stream is the transcriptome, or RNA expression data, which is looking at the levels of RNA messages that are transcribed from DNA and may be resident in cells at any time. The farther future might include a comprehensive profile of RNA messages circulating in cells at any given time. This information is of increasing interest as the capability now exists to peer into cells in real-time to assess whether certain genes are expressed or not. Similar to the transcriptome, the metabolome provides a snapshot look at the levels of metabolites such as sugars and fats that are resident in cells. Metabolites are the chemical signatures (hormones and other signaling molecules) left behind in cellular processes, and are useful in the analysis of many things such as whether different aspects of the cell are functioning normally. 

The microbiome (the ten times the number of human cells (1–2 grams) that are carried on and in each person as bacteria) is an important emerging health data stream. The recent completion of the Human Microbiome Project has helped to categorize normal bacterial populations and their role in disease development, drug response, and nutrient synthesis [9]. Other data streams, the environmentome and diseasome, are similar concepts of overall profiling to elucidate the baseline status and response of individuals in specific contexts. The environmentome is a measure of the impact of the external environment and an individual’s ability to process toxins, and the diseasome is a measure of an individual’s risk for developing different diseases in the future. These newer health data streams ranging from genomics to the diseasome are a complement to the traditional health data streams of personal and family health history, blood test data and other laboratory results, and prescription histories. 

These diverse health data streams are not just important in isolation, but are being applied together in a systems approach suited to the complexities of the underlying biology. A standard example is researchers looking at genomic profiles and epigenetic changes together with cellular expression information to understand how pathology develops at the molecular level, for example in the cases of autism and rheumatoid arthritis [10,11]. These data streams are interesting not only for research, but should also be deployed in clinical care. New health stream data will need to be collected, integrated, and managed in clinical systems, with the relevant information aggregated in ways tailored to practical implementation.

The vast amounts of data already being generated by current medical information practices (e.g., medical records and imaging data) and even more so in preventive medicine (e.g., genomic files, metabolic profiles, quantified self-tracking data, *etc.*) raise the important role of information management tools. The first tier is electronic medical records (EMRs), which could become more detailed over time. The current focus on personal and family health history, prescription records, and current diagnosis and treatment details could be expanded to include predictive risk-assessment modules at the front-end to facilitate the practice of preventive medicine by integrating the relevant health information streams depicted in Figure 1. The second tier is secure cloud storage systems with different levels of permissioning access for the various data streams. Another tier is machine learning and other algorithms to run on top of the big data to search, access, and aggregate meaningful data patterns, and translate them into actionable information and real-time personalized recommendations.

### 2.3. Participant-Centric Action-taking

Now moving to the middle of the diagram, the individual is at the center of action-taking related to health and health care. There are three progressive lines of defense around the individual. First is the continuous health information climate of always-on self-tracking devices and smartphone applications. This technology blanket may increasingly provide automated digital health monitoring, data collection such as baseline measures and variability norms, and ambient behavior management suggestions and other real-time personalized recommendations. Already self-tracking gadgets and applications are proving to be a mainstream phenomenon as 80% of U.S. Internet‑connected adults have searched for health information online [12] and thousands of consumers have snapped over 7.7 million food diary photos with ‘The Eatery’ smartphone application [13]. The second line of defense around the individual is collaborations with health advisors and peers who have interests in similar conditions. The expanding ecosystem for a more proactive approach to health includes interest groups like health social networks, crowdsourced studies, and the Quantified Self community. New categories of health service providers, potentially compensated with Health Savings Account (HSA) dollars or other novel payment structures, include health advisors, wellness coaches, preventive care providers, boutique physicians, and genetic counselors. Next-generation software solutions in the form of more tightly-integrated personal electronic health records are also part of this second layer of defense. Finally, the third line of defense around the individual is the public health system where, after wellness maintenance resources have been exhausted, the deep expertise of traditional health professionals is appropriately and critically valuable for disease and trauma diagnosis and treatment. 

#### Mental Performance and Cognitive Acuity is the New Health Frontier

In the near-term, mental performance could emerge as the new health frontier, a critical component of the notion of overall health. Much like personalized genomics has helped to destigmatize a variety of physical disease conditions, tracking tools could do the same for mental health. Cognitive performance could come to be seen as a performance optimization activity with tools available for its improvement, rather than as a deterministic definer of identity and possibilities. With personalized genomics, we see that we are not in world as depicted in the dystopian science fiction movie Gattaca where some individuals are genetically perfect and others are not. In our world, it is likely that every individual is at higher than average risk for at least one of the top twenty common disease conditions such as heart disease, diabetes, and cancer. This knowledge, together with the understanding of the non-causal responsibility of the individual has helped to destigmatize disease and impel focus instead on cures, and this attitude could persist into mental health. 

Emerging quantified data streams could be helpful in elucidating the mental health of both individuals and populations. The U.S. National Institutes of Health (NIH) have estimated that 26.2% of Americans ages 18 and older, one in four adults, suffer from a diagnosable mental disorder in any given year [14]. In addition to stigmas surrounding seeking mental health assistance, costs are high, and options are not well-known. A shift to the positive positioning of mental performance optimization techniques rather than disease cures or ‘seeking help’ may cause more people to investigate solutions. Additionally, a number of new health data streams may be extremely revelatory such as measuring baseline and variability in individual and population levels of biophysical chemicals like cortisol (related to stress), oxytocin (related to feelings of connection), and dopamine (related to the ability to focus), and quantified assessments of qualities such as empathy, loneliness, happiness, and fulfillment. With the presence of technology tools such as the therapeutic intimacy of the mobile phone, mental performance assessment and optimization could be extended quickly to the vast majority of the population. Recreational voice-based chatting with Siri, an intelligent personal assistant on the iPhone, is possibly an early harbinger of what may become more elaborate personal virtual coaches delivering real-time mental performance optimization capabilities. Some efforts already underway in mobile mental health improvement include mood tracking smartphone applications like MoodPanda, mood charting per text reminders [15], the myCompass program for mild-to-moderate stress, anxiety, and depression management [16], and an application for better heart health through breathing exercises and better emotion management through mood capture and intervention [17].

### 2.4. The 80% of a Condition’s Life Cycle While It Is still Pre-Clinical

Critical to the broader concept of health and health care is the notion of wellness and prevention. The tools, capability, and understanding are increasingly available to identify conditions ahead of time and take measures which could ultimately result in prevention. Access to preventive medicine tools by the largest possible segment of the population is critical. Preventive medicine is inherently democratized with the individual as the center of action-taking with free or cheaply available mobile phone applications, online personal health records, and other increasingly inexpensive or sponsored self-tracking and monitoring solutions. The most successful initiatives for engaging individuals in the health context so far have had value propositions that incorporate first and foremost, personalized recommendations, and secondarily, social interaction, gamification, attractive data visualizations of contributed information, and other modern techniques to make using technologies fun while simultaneously achieving behavior change goals [18]. The emerging preventive medicine ecosystem is articulated in Table 1. 

#### 2.4.1. Concept of Health, Service Providers, Remedy Providers, and Research Conduct

As seen previously in Figure 1, the first step is noticing that the definition of health and health care is expanding to mean wellness maintenance and condition prevention as opposed to just the cure of illness. One indication of the growing ecosystem in health is the increasingly-used distinction business are making between wellness and medicine. Service providers too are changing, with a multiplicity of health advisors, genomic counselors, health maintenance specialists, concierge physicians, and targeted therapy providers such as personalized cancer genomics services CollabRX (Palo Alto, CA, USA) and Foundation Medicine (Cambridge, MA, USA) to supplement and off-load from the traditional public health system. Remedy providers are expanding too, from pharmaceutical companies to more of a focus on supplementation, stress reduction, and other preventive treatments. Health engagement platforms like Massive Health with the previously mentioned food photo-journaling application, The Eatery [20], are becoming a node in the landscape, offering an effective mix of quantified self-tracking data collection tools, lightweight social networking interaction, and gamification rewards, all of which combine to influence behavior change. The company has reported that consumers improve the healthiness of their eating habits within one month of using the application, however these reports are not without criticism. Health research too is shifting, from formerly being conducted primarily in academic settings, to now being executed in a variety of crowdsourced cohort programs, health social networks, and other forms of CRO 2.0, the next generation of the Contract Research Organization (organizations that coordinate the operation of clinical trials) [18].

**Table 1:** The 80/20 Model: Addressing and eliminating conditions while still pre-clinical.

*Category*	Pre-Clinical (80%)	Clinical (20%)
*Concept of health*	Wellness maintenance, prevention	Illness cure
*Service providers*	Wellness: Health advisors, wellness coaches, genomic counselors, prevention specialists	Medical: Public health system
*Remedy providers*	Health engagement platforms, health social networks, peer collaborators, supplementation treatments, medical tourism	Pharmaceutical companies
*Research conduct*	Crowdsourced studies, health social networks, CRO 2.0 ^a ^[18]	Academia
*Financial models*	HSA, ^b^ out of pocket	Professional payers, insurance
*Privacy and security*	Cloud, PHRs ^c^	Physician office, paper files
*Legislative influence*	Patient Advocacy Groups	Medical professionals
*Regulation and oversight*	Portable consent [19], IRB 2.0 ^d^	Institutional IRB

^a ^CRO: contract research organization; ^b ^HSA: health savings account; ^c ^PHR: personal health record; ^d ^IRB: institutional review board.

#### 2.4.2. Financial Models and Economics

Economics is one of the most important components in enabling a shift to preventive medicine. The health services industry could be another institutional juggernaut to crumble, following publishing, music, and other industries. Incumbents have little incentive to change given that they are the few organizations making the most money, but countervailing forces could impose. Widespread adoption of HSAs by employer-funded health plans is a favorable step since price rationalization has been pushed directly to the consuming party. It is nearly impossible for consumers to obtain the final out-of-pocket cost for health services ahead of time, and this is a significant pain point in the system that could eventually force innovative solutions. It is clear that the ecosystem of funding sources and models for health service economics needs to be expanded. Innovative health economics models could be useful both unitarily (benefiting consumers of specific solutions) and systemically (facilitating pervasive institutional price and cost rationalization and validating the link between cost and benefit).

Health is one of the few industries for which cost and price is hidden and in many cases unknown, and has enormous variance as each payer negotiates separately with each service provider. Greater price rationalization and tighter linkage between service recipient and payer could help to make newly emerging health services models more democratic and avoid the problem of new monopolists arising in the ashes of the old if the systemic incentives have not changed. There is already a move away from exclusive dependence on insurance companies and other professional payers to include HSAs, out-of-pocket payments, and other financing models such as specialized consumer health credit card programs like CareCredit. 

Other innovative health economics models are nascent but are already starting to have a positive impact on the administration of health care and health research funding. Some non-traditional sources of funding include patient advocacy groups, more-radically oriented research foundations, social venture capital, crowdfunding, and self-funding [21]. Crowdfunding is a particularly interesting new financial tool made possible by large-scale crowdsourced Internet models. Crowdfunding is defined as appealing to many individuals via the Internet to contribute small amounts to fund targeted projects of interest. Some examples of crowdfunding websites are Kickstarter (in a famous case raising $10 million for the Pebble smartwatch project), Petridish, and RocketHub, and emerging health-specific crowdfunding sites like MedStartr. One or more dedicated health research crowdfunding sites could be quite timely, allowing both professional researchers to supplement their traditional grant-based funding sources, and participant-organized studies to find funders for special-interest and preventive medicine health projects more expediently. Both the public and patient advocacy groups have a preference for non-pharmaceutical remedies, and an interest in investigating their efficacy. Health research study crowdfunding efforts could be forward-integrated into health research collaboration communities to pre-fund studies where there is market demand and facilitate recruitment, and backward-integrated to automatically support individual participation in crowdsourced studies. Funding could become a recruitment tool and recruiting could become a funding tool. 

#### 2.4.3. Privacy and Security, Legislative Influence, and Regulation and Oversight

Concerns over security and privacy remain tantamount. Here too there is innovation to facilitate the transition to the preventive personalized health of the future. Health privacy models are being reinvented with users storing their data in PHRs (personal health records) in the cloud and controlling access through different permissioning tiers, as opposed to the old model of patient data residing in isolated physician office storage systems. The new era of big health data requires new privacy models, and also engenders new models of political influence. Patient advocacy groups may start to have increased lobbying power and legislative influence as more individuals, finding out that they are at risk for certain conditions, join advocacy groups and support collaborative research and other preventive and investigative activities. Alternatives to regulatory and oversight models are starting to emerge as well. One example is the idea of having a portable consent for personal data contribution to studies [19], and the possibility of meeting the traditional responsibilities of IRBs (institutional review boards), ethical oversight and liability-grounding, through separate mechanisms [18]. 

Another dimension of regulatory shift that may slowly ebb into existence is regarding classifications and approvals. More granular regulatory definitions to support and stimulate the new generation of self-analysis tools are required. One example is the legal distinctions regarding what constitutes a medical diagnosis. Technically in the U.S., only physicians are allowed to make a diagnosis, however, the ‘return of research data’ may look similar to a diagnosis if the data are presented in certain ways. This could be codified into acceptable legal practice or defined as a new category like ‘pre-diagnosis’ which could be acceptable from a legal, ethical, and regulatory perspective and help to foster greater growth in preventive medicine assessment technologies. An increasing number of quantified health‑tracking and detection gadgets are coming to market and currently need different kinds of regulatory approval. One solution in the U.S. is to seek 510(k) clearance and a CLIA waiver for devices such as a recently launched influenza detection panel from health consumables provider Becton Dickinson [22]. Another solution is to go to market more quickly in countries that are providing fast-access medical device registration as a specialization policy, for example Singapore, Hong Kong, Malaysia, Israel, Colombia, and Costa Rica. Global regulatory consultancies such as the Emergo Group (http://www.emergogroup.com/) provide strategic services regarding medical device market entry. In more burdensome regulatory regimes such as the U.S., preventive medicine efforts could be spurred with a revised stratification of graduated approval levels that allow the new class of low-risk non-invasive personal health assessment tools to come to market more expediently. 

## 3. The Realization of Health 2050: Preventive Medicine

### 3.1. Personalized Typing

**Figure 2:**
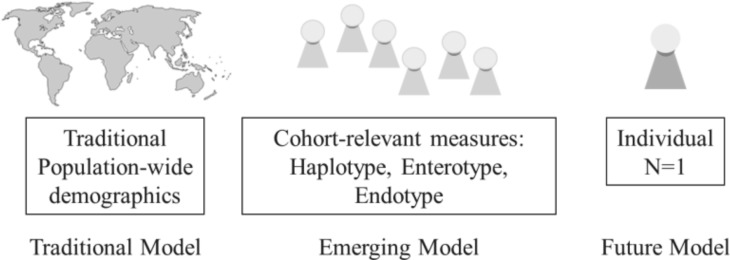
Classification models in personalized medicine.

Critical to Health 2050: Preventive Medicine is addressing conditions during the 80% of their life cycle before they become clinical. In the past, there was little alternative in many cases but to conduct disease treatment based on phenotypic presentation as opposed to underlying disease mechanisms. Disease classification has always been challenging, especially given the realities of biological heterogeneity where it can be difficult to distinguish between one disease presenting in different ways and the same symptom being generated by different pathologies. One important question is how to appropriately group individuals into useful categories, and the basic models for doing so are depicted in Figure 2. 

It is now possible to expand beyond broad population-level distinctions such as demographics and socio-economic indicators as the main classification parameters, and include measures of greater health relevancy to classify individuals into specialized cohorts. One example of cohort-relevant measures is identifying those at high risk for type 2 diabetes based on genetic risk, hemoglobin A1c levels [23], weight, BMI (body mass index), family history, and smoking status, as opposed to not having quantitative measures like genetic polymorphisms and blood risk data previously. Eventually, this may lead to being able to focus precisely on the unique health complexity of individuals. More granularity in classifying individuals into cohorts of health relevancy can also be called typing, extending models like the four blood type groups into other areas. Some examples are genetic haplotype group, enterotype, and endotype.

#### 3.1.1. Genotyping and Haplotype Groups

In genotyping, it was soon realized that single mutations or SNPs do not account for much of disease causality, perhaps only up to 5% [24]. However, it is currently thought that structural variation (chunks of sequences that are deleted, repeated, transposed, appear in another location, *etc.*), epigenetics (genetic changes that develop during an organism’s life), and SNP mutations could all be investigated together within the ensemble of haplotype groups to explain more of disease causality. A haplotype group is a collection of alleles that are transmitted together and may relate to different health characteristics or disease risk profiles. At present, haplotype groups are being assessed for diabetes [25], obesity [26], hypertension [27], and immune disease (looking comprehensively at the genes in the major histocompatibility complex (MHC)) [28].

#### 3.1.2. Enterotyping the Microbiome

Typing is also a technique used in the emerging health data stream of the microbiome. The microbiome provides numerous benefits without which we could not survive, including food digestion, vitamin synthesis, metabolic regulation, immune system regulation, and pathogen resistance. Research is now beginning to understand the important role that the microbiome has in disease development, drug response, and nutrient synthesis. Early work, not without controversy, has suggested that humans may be classified into one of three microbiome enterotypes [29]. These enterotypes are Bacteroides, Prevotella, and in some cases, Ruminococcus [30]. The enterotype indicates which nutrients an individual may be better at synthesizing, for example, B2, B5, C, and H for those with a higher prevalence of Bacteroides, and folic acid and vitamin B1 for those with a higher prevalence of Prevotella [31]. Enterotype might be predictive of diet and disease—a higher prevalence of Bacteroides is linked with a diet high in fat and protein, and greater risk for obesity and metabolic disease, while a higher prevalence of Prevotella is linked with a diet high in carbohydrates [31]. Two recent projects have been launched to target the consumer market: the ‘Microbiome Profiling Response to Probiotic in a Healthy Cohort’ from DIYgenomics and Second Genome, and the ‘What would you do with your microbiome sequence?’ project from the Quantified Self and Pathogenica.

#### 3.1.3. Endotyping Asthma

Endotyping is the name of the similar typing technique used in asthma, where subpopulations of individuals with the condition are defined based on molecular, functional, or pathobiological mechanisms. The approach allows individuals to be grouped into different treatments more effectively. Asthma is broadly characterized by variable airflow obstruction, bronchial hyperresponsiveness, and inflammation, however in any patient different symptoms may be predominant or absent. At least two groups have proposed different endotype classification systems which could progress towards a medical standard. Some of the endotypes suggested relate to distinctions between eosinophilic, neutrophilic, atopic, non-atopic, early onset, late onset, and aspirin-sensitive or exercise-induced asthma [32,33]. One challenge is that the asthma endotypes are potentially less precise in assessment than genetic haplotype groups or microbiomic enterotypes as they may be based on a wider range of parameters including clinical characteristics, biomarkers, lung physiology, genetics, histopathology, epidemiology, and treatment response. 

### 3.2. Participatory Health Efforts

The individual is a critical component in realizing Health 2050 in the expanded concept of health and health care (Figure 1) and in the 80/20 notion of addressing conditions while they are still pre-clinical (Table 1). Participatory health [21] or participant-centric initiatives [34] is a term indicating the shift towards the empowerment of the individual and indicates that the individual is at the center of action-taking related to health. One of the first uses of the idea of participatory health was in 2008, as one of several terms being used interchangeably, including Health 2.0, Medicine 2.0, and eHealth. The term meant “*use of a specific set of Web [2.0] tools (blogs, podcasts, tagging, search, wikis, etc.) by actors in health care including doctors, patients, and scientists, … in order to personalize health care, collaborate, and promote health education [35]*.” The individual was now as equally disposed as professionals to action-taking through social media to personalize health care. From this foundation, a more significant move came in 2010 when the Society for Participatory Medicine, itself a new organization, declared that “*Participatory Medicine is a movement in which networked patients shift from being mere passengers to responsible drivers of their health, and in which providers encourage and value them as full partners [36]*.” Individuals were now seen as instigators and drivers of their own health in all respects, not just through social media. 

Since then, a wide ecosystem of participatory health efforts has begun, offering individuals diverse participation possibilities from light to intensive engagement as depicted in Table 2 [37]. Such participatory health efforts include social media, health applications on the mobile phone, personal electronic health records, health social networks, direct-to-consumer tests such as genomic and blood tests, and crowdsourced health collaboration and experimentation communities. 

**Table 2:** Participatory health activities ranging from light to intensive engagement.

(Light)		Level of Participant Engagement		(Intensive)	
Social Media	Mobile Phone Health Apps	Personal Health Records (PHRs)	Health Social Networks (HSNs)	Consumer Genomics	Crowdsourced Health Studies

#### 3.2.1. Social Media

Social media refers to using the usual lightweight Internet-based platforms for online search, messaging, media consumption, and social networking for health-related purposes. Some of the standard social media include blogs, Twitter, Facebook, Google+, wikis, search, and video sites. Social media serves as an innovative, valuable, and increasingly standardized communication tool for health information dissemination, real-time feedback, multi-party interaction, and other uses. A 2011 Pew Internet study found that 80% of Internet users look for health information online, 27% of U.S. Internet users had tracked health data online, and 18% had sought to locate others with similar health concerns via the Internet [12]. These statistics suggest that health empowerment and action-taking is becoming a mainstream behavioral norm as opposed to the limited activity of a small group of health enthusiasts. 

#### 3.2.2. Mobile Phone Health Apps

The importance of mobile phone health applications cannot be overemphasized in realizing Health 2050: Preventive Medicine. The number of worldwide smartphone users is expected to exceed one billion by 2013 [38]. Application downloads grew explosively from 300 million in 2009 to five billion in 2010, and over 7,000 apps are health-related [39]. The principal consumer uses of smartphone health applications are for education, information, and self-tracking of diverse physical and mental conditions. 

Mobile platforms and health applications are also useful to medical professionals for real-time communication, information access, and telemedicine. 81% of U.S. physicians are using smartphones [40], and 62% of those surveyed in one study are using the iPad professionally [41]. The hurdles are not technical but structural, as of March 2011, only 12 U.S. states were offering reimbursement for telemedicine services (e.g., telephone, email, video consultation), and at lower reimbursement percentages than traditional in-office visits [42]. Now more payers and states are starting to approve telemedicine reimbursement and Health 2.0 companies such as HealthTap are launching sleek smartphone applications for private medical consultation [43]. This could be a floodgate of cost savings for the industry and a much better use of physician time as it is estimated that 70% of physician consultations could be handled by phone [44]. Regarding social media, one study found that physicians are using social media, 87% for personal use and 67% for professional use [45], while another found that 20% of physicians emailed with patients and 6% communicated with them through social media [46], mostly preferring to decline Facebook friend invitations, for example. 

Aside from consumers and medical professionals, health research is another beneficiary of the new era of social media and mobile phone apps. The sheer number of mobile phone users has already offered the possibility for research efforts to scale up by at least an order of magnitude. In one example, thousands of worldwide study participants (4,157) were recruited within months, as opposed to the few hundred that could be targeted previously on a more cost-limited basis [38].

#### 3.2.3. Personal Health Records (PHRs)

Personal health records (PHRs) are medical records owned and administered by patients rather than health care professionals. They may contain the same information as traditional medical records such as blood type, family and personal health history, and prescription information, as well as new kinds of data like personal genome profiles. PHRs are typically online, with patients administering the records and granting specific permissions to different health care providers as needed. PHRs are a key step in empowering health self-management as we can have a more active role in understanding, accessing, maintaining, and sharing our personal health information, and in coordinating and participating in our own health care. One health provider found that PHR users were 68% better at following up on recommended care than non-PHR users [47], indicating the potentially useful behavioral influences of PHRs. Additional aspects of PHRs regarding the integration of information from various health data streams were discussed earlier in Section 2.2.

#### 3.2.4. Health Social Networks (HSNs)

Health social networks (HSNs) are online health interest communities where individuals may find and discuss information about conditions, symptoms, and treatments, provide and receive support, enter and monitor data, and join health studies [48]. Health social networks cater to both the general public (e.g., MedHelp, PatientsLikeMe, and DailyStrength) and specific groups (e.g., Tudiabetes, Asthmapolis). They may be consumer-focused or physician-focused (e.g., Sermo, Ozmosis, and RadRounds) [21]. More recently, drug health social networks have arisen such as Treato and eHealthMe to find out how other patients have responded to specific medications and therapeutics. The shared aggregated data of individuals contributing to health social networks creates a valuable public good which can benefit populations on the whole. 

#### 3.2.5. Consumer Genomics

Consumer genomics is part of the more general trend of health-related tests being available directly to consumers. Unbeknownst to many people, consumer blood tests, for example from DirectLabs and the Life Extension Foundation, have long been available directly to consumers via the Internet without a traditional doctor’s office visit. Consumer genomics had a big impact when test kits were made available directly to consumers in the 2007 time frame and some medical professionals raised concern. After ongoing regulatory involvement, consumer genomics tests continue to be available in most industrialized countries from a variety of providers (e.g., 23andMe, deCODEme, Navigenics, and Pathway Genomics, though the latter two require a physician consultation) and have approximately 150,000–200,000 total subscribers [49]. 

Consumer genomics is notable in that this was one of the first times that significant amounts of health-related data became available directly to individuals without the mediation of medical professionals. Despite ongoing concerns regarding the utility and interpretive validity of personal genomic information [50], the advent of the consumer genome was an important milestone in individual empowerment towards health data, and engendered a critical maturation point in the mindsets of subscribing individuals, particularly regarding access and ownership rights to the health data of the individual. Other efforts related to health data ownership and sharing have been inspired such as “That’s My Data!” where patients share their genetic data with researchers in exchange for open access to the results [51]. The current status of consumer genomics is that there is a perception that probabilistic risk information for health conditions remains difficult to make actionable, while drug response genomics is increasingly useful. As of July 2012, the FDA has validated genetic testing for over one hundred drugs [52].

#### 3.2.6. Crowdsourced Health Studies

Crowdsourced health studies and quantified self-experimentation projects conducted individually and in groups are emerging as an important complement to traditional clinical trials and other established mechanisms of health knowledge generation. In these studies and projects, participants are crowdsourced via meetup groups or the Internet, *i.e.*, recruited in vast open calls using social media and other techniques allowing individuals to self-select participation. Crowdsourced health research studies may be organized by traditional institutional researchers, non-professional researchers, or by the study participants themselves. Integrating self-tracking device data and crowdsourced health experimentation results into personal electronic health records for an overall picture of preventive health is an important medical challenge. There are many potential benefits to crowdsourced research studies. They are seen as complementary to traditional studies where the Internet serves as a barometer for surfacing salient information via crowdsourced health studies with preliminarily interesting findings that could then be further investigated in traditional studies [21]. Crowdsourced studies are the venture capital round of health research studies. 

### 3.3. Era of Big Health Data

Big data is an important contemporary trend, comparable in impact to the personal computer or the Internet, that is reshaping the employment economy and many industries including health. Data is growing at 50% a year, or more than doubling every two years [53]. Some of the challenges are that it is not yet possible in all cases to determine which data are of relevance, how much data should be stored, and how it should be made accessible. ‘Big data’ refers to the collection of voluminous amounts (e.g., petabytes and exabytes) of a variety of unstructured and semi-structured data that is now possible, cheap, and occurring in most sectors of the economy. Analyzing the data (analytics) and information visualization (data viz) therefore become immediately critical for churning through the large data sets to produce meaningful insights. 

#### 3.3.1. Search and Social Media Aggregation of Health Information

There are a number of big health data applications already in use. One of the most basic is scraping Internet content for health information. Search companies Google and Yahoo track trends in search data on health topics. Some of this information has been turned into projects such as Google Flu Trends and a Yahoo research effort regarding information needs when experiencing grief [54]. However, one analysis noted that these methods (e.g., analyzing search data) may be problematic—in the flu case, search results were predictive of flu symptoms, but not actual cases [55]. Beyond aggregating the behavior of individuals on the Internet with regard to health to assess the type and level of concerns (using search keywords as a proxy), public health surveillance is another application area for big health data. 

In public health surveillance, researchers process news and social media for information insights. One example is the Health Map Data project, with over six years of worldwide data concerning infectious disease outbreaks. The information is made available via a mobile phone application, Outbreaks Near Me. The method was used to successfully elicit epidemiological patterns in a cholera outbreak in Haiti [56]. A new effort by the group, Flu Near You, is intended to track the patterns of how the flu develops and spreads across communities. Other similar public health surveillance efforts include Sickweather, tracking disease through social media chatter, and Transform Health, intending to provide real-time maps of human health and illness with mobile phone applications. Geolocation-aware mobile crowdsourcing applications may also facilitate public health surveillance, disaster reporting, and ongoing response and information collection for any variety of health-related issues [57]. Underlining the importance of effective lightweight applications for public health surveillance, a government-sponsored hackathon challenge took place in the U.S. in June 2012. The Now Trending Challenge led by the U.S. Office of the Assistant Secretary for Preparedness and Response (ASPR) sought web-based applications using Twitter to identify trending illnesses [58]. Like the flu search trends projects, public health surveillance data projects will need to be assessed for accuracy. 

#### 3.3.2. Using Big Health Data for Preventive Prediction

Big health data applications pertain to both institutional and crowdsourced efforts. Health service providers and insurers are mining biomedical information as a strategic imperative in running their operations. One example is a health data analytics company, OptumInsight, arising from a large U.S. health care company, UnitedHealth Group. Big data analysis techniques were applied to 90 million health claims and associated data during the 1993–2012 period to make predictions about illness occurrence and treatment needs in other similar patients. Even a few simple factors such as weight, BMI, smoking, and family history were shown to be predictive for diabetes. 

Big data together with crowdsourcing and the emergence of new models such as prediction markets (a mechanism for capturing group opinion) is the Iowa Electronic Health Markets. At this website, individuals may register their opinion in real-time regarding the scope, spread, and duration of epidemics and other health events. A related site, Kaggle, offers the ability to post and compete in data science challenges. Crowdsourced participants analyze large data sets to predict hospital admittance rates, consumer behavior, and sales forecasts. The Kaggle data science projects have just begun and results are not available yet, but crowdsourcing has proven successful in other biological data challenges like FoldIt, a computer-based game for tackling the complexities of protein folding. Gamers took just days to solve a monkey virus retroviral protease structure [59] and found an 18-fold-more-active version of a model enzyme [60]. Another project used mobile phones to collect large amounts of crowdsourced health reporting data in a participatory epidemiology project [61].

Big health data applications are important in the realization of preventive medicine at both the macro and micro level. At the macro level, they provide the capability to track health-related issues at the vast scale of worldwide populations in low-cost ways. This is useful both directly for planning and immediate response to outbreaks and other situations, and indirectly in creating a large longitudinal dataset of health-related information as a public resource. At the micro level, the passive data collection activities of always-on self-tracking devices can help individuals to establish both quantitative and qualitative baseline and variability understandings of a variety of health-related issues and behaviors, and the technologies can make ambient inquiries and subtle suggestions, for example querying as to why someone may have less physical activity this week than last. 

### 3.4. Change in Philosophical Mindset and Other Qualitative Shifts

The realization of Health 2050: Preventive Medicine is both quantitative and qualitative. Not only are there new advances in the quantitative ways of science, but the qualitative meaning of the new tools and knowledge is equally important. There is a subjective dimension of concerns such as mindset, experience, emotion, ethics, values, and culture at the levels of the individual, family, community, and society that is outside of the realm of reason, science, a system, or some other form of objective truth. A simple example of a developing shift in a subjective domain is the paradigm of the old thinking ‘My health is the responsibility of my physician,’ being replaced by the new thinking that ‘My health is my responsibility, and I have the tools to manage it.’

#### 3.4.1. Overview of Participatory Health Communities

Participatory health studies in crowdsourced cohorts, health social networks, and *n* = 1 self-experimentation communities is a growing trend. As of July 2012, one high-profile health social network, PatientsLikeMe, had over 157,000 community members participating in 1,000 conditions. Consumer genomics community 23andMe had over 150,000 subscribers [49]. Genomera, a personal health collaboration platform where community members (both professional researchers and citizen scientists) operate studies had over 25 studies listed and 800 community members ready to participate in crowdsourced studies with genotypic and phenotypic information. The Quantified Self community is a fast-developing movement where both health enthusiasts and diagnosed patients meet in an environment of trust to share the quantified self-tracking projects they have been doing in the format of monthly show-and-tell groups. As of July 2012, the Quantified Self community had 65 worldwide meetup groups with thousands of participants after only four years of existence, and a third annual conference planned for September 2012.

A number of forces are uniting to facilitate participatory health including the emergence of trust and empowerment in Internet-based social networking communities together with low-cost newly available technology like genome sequencing and bio-monitoring applications and devices. How an individual understands his or herself in regard to health and health research is changing. In the past, *n* equaled someone else, the population average, which may or may not apply on an individual basis; now, ‘*n* = me’ and the information applies directly [62]. Further, there is the idea of ‘*n* = we’ developing as self-experimenting, self-empowered individuals come together in health collaboration communities like the Quantified Self, DIYgenomics, PatientsLikeMe, and Genomera to make their *n* = 1 discoveries less anomalous, statistically significant, and scientifically rigorous [62]. The definition of what it is to be a biocitizen in the modern world is changing, and starting to include data-sharing, study participation, and more proactive health self-management and responsibility-taking.

#### 3.4.2. Motivations of Crowdsourced Study Participants

Uncovering the motivations and experience of individuals engaged in participatory health initiatives is one way to understand the qualitative shifts occurring in Health 2050: Preventive Medicine, and suggests that the phenomenon is not restricted to health enthusiasts but rather extends to the population more generally. In one of the first studies where participants organized a research effort and published their results, personal statements were specifically included as a qualitative dimension. The study examined genetic variation, vitamin B serum levels, and the impact of the passive versus the active formulation of vitamin B supplementation, and found that baseline blood levels were more likely to be out-of-bounds for those with a genetic mutation and that a simple drugstore multivitamin was successful for most in quickly remedying the condition [63]. 

The personal statements collected as part of this DIYgenomics vitamin B study addressed motivating factors for participating in the study, reaction to study results, and resulting behavioral changes. Participation motivations included wanting to understand how personal genetic profiles related to serum vitamin levels and interventions, wanting to determine generally if there was a benefit to taking vitamins, and exploring how to use personal genomic data to make positive health changes. Reactions to the findings were noting that not everyone responded in a similar manner to the same intervention, disappointment at the lack of information regarding which vitamins might be appropriate for different genotypes, and surprise at how quickly and effectively the interventions worked. Regarding ongoing behavioral change, nearly all participants noted intended modifications, specifically continuing to take the supplement that was most effective on an individual basis, greater adherence to supplementation programs, and interest in further experimentation. Two years after the study completion, several study participants continue to incorporate their personalized experimental results in daily regimens. Perhaps the most interesting result of psychological and philosophical importance reflected in the personal statements was surprise at the depth of the personal impact even in a fairly simple study. Participants noted that published study results from traditional clinical trials were not at all necessarily the best intervention on a personal basis, and commented on the value of the study results in ongoing personal health self-management. 

#### 3.4.3. Quantified Self Study: Are New Forms of Knowledge Being Generated?

A next level of investigating the qualitative shifts in Health 2050 towards personalized preventive medicine is asking more probing philosophical questions about the knowledge-generation that is taking place. The first point to examine is whether, in fact, new kinds of knowledge are being derived from personal experimentation and group collaboration, or if it is the same kind of knowledge, just being derived differently. The second point is how to characterize and describe the knowledge that is being generated in participatory health efforts. Third is to understand how the knowledge is being understood, appropriated, and made actionable by individuals, and what this means from a personal and societal perspective for the emerging biocitizen. 

A study examining these questions is the DIYgenomics epistemology study: ‘Knowledge generation through self-experimentation.’ One outcome from the study is to be able to develop an epistemology of citizen science that can provide a structure and context for participant-derived health knowledge, and more broadly-articulate, formalize, and validate knowledge derived from any citizen science project. A central hypothesis is that knowledge derived from self-experimentation, or even from personal genomic data or self-tracking gadgets, is qualitatively different than traditionally obtained health knowledge. First, in the past, health data were nearly always mediated, often paternalistically, conservatively, and normatively, by medical professionals. Second, the type of data is new—previously data was not typically sought or relayed unless it pertained to a diagnosis or treatment in the context of clinical conditions and their remedy. Now, in the always-on health information climate, reams of potentially irrelevant data are collected passively and must be orchestrated and reviewed for personal health relevance in the complex game of preventive medicine where the connection between individual data points and micro-behaviors is not obviously correlated with diagnosed conditions or immediate action-taking. Third, as seen in the personal statements of participatory study participants above, today’s health knowledge is fundamentally different in its level of personal applicability and relevance. When information is obtained directly through self-experimentation as opposed to from a doctor who is relating published study results that have general effectiveness at the population level but not at the individual level, the meaning is significantly different, and translates much more expediently to behavioral change, and the empowerment of the biocitizen.

#### 3.4.4. Quantified Self Study Results

In the DIYgenomics knowledge generation study, participants are asked to complete an online questionnaire regarding their quantified self-experimentation projects in any area of activity. Projects are often regarding health, time-management, stress-reduction, nutrition, exercise, mood, and sleep optimization. The preliminary results are already informative. Self-experimentation projects typically fall into two categories. Most projects have been fairly short-term (e.g., on average two months) for the purpose of addressing a particular issue, often with the desired outcome being a quality-of-life improvement. The other category of projects is periodic longitudinal data collection for the purpose of establishing and monitoring baseline norms, and conducting ongoing health management and optimization. There are three common and notable aspects about the quantified self-experimentation efforts. First, experimenters tend to define a clear outcome at the beginning (e.g., improve sleep). Second, they iterate rapidly through many different interventions, and possibly approaches, whilst tracking the effectiveness of each. Third, experimenters generally achieve some sort of result that either solves the issue or provides some other endpoint or moment for recasting the experimentation. The tone of participant experience narratives is practical rather than introspective, and, having resolved an issue, a participant may even forget what it was like having that issue.

Many participants in quantified self projects are aware of the potential limitations of their efforts and try to adjust for this. In one example, an experimenter was particularly rigorous with dosages and times of testing, much more so than he would have been in an institutionally-run study, due in part to the self-funded nature of the experiment. Another tried to be as scientifically accurate as possible in tracking and experimental methods so that the results would not be anecdotal. Experimenters found the accuracy of data results to have high fidelity and personal meaning. Participants noted that more data, better tools, sharing with others, and lower costs for blood tests and other measurement validation would be potential improvements to their experimentation capability. Some of the outcomes for experimenters were one individual finding that optimal vitamin consumption varied depending on the time of year, another that niacin contributed to managing cholesterol levels, and another that diet specificities may be contributing to acne development. Others succeeded in improving sleep quality, one by finding that caffeine intake was the most critical influencing factor, and another that slightly inclining the mattress resolved the issue as opposed to many other experimental interventions related to caffeine and alcohol consumption, light and television exposure, and the timing of going to bed. 

#### 3.4.5. Participatory Health Pioneers Are Defining the Preventive Medicine Mindset

While criticism has been levied against quantified self-experimentation and crowdsourced health study participation as being the special-interest activities of a small minority of those particularly, and potentially obsessively, interested in health tracking and improvement, it can be argued that these pioneers are critical in facilitating the widespread realization of preventive medicine. It is the ‘Wikipedia-ization of health’ as a small number of contributors join to create a public good of extensive and universal value. In technological and social movements, early adopters not only pave the way at the practical level, innovating tools and experimental processes so that they can be made cost-efficient and codified into effective means for attaining results, but also at the qualitative level. Early adopters help to make new ideas and techniques known as initially being strange and preposterous, but then more commonplace as a sensibility and maturity develops, and value propositions become defined to different audiences. Self-awareness, self-tracking, monitoring, experimenting, and action-taking are critical components in the preventive medicine movement, and early adopters, health hackers, and gene geeks are expected to innovate at the radical edge as the first step towards mainstream adoption. The full path to the realization of the personalized preventive medicine of the future is just starting to be defined, and the way forward will likely be elucidated by innovators, both institutional and individual, and then expanded as other groups see and implement the value for themselves.

### 3.5. Limitations

There are a number of reasons that Health 2050: Preventive Medicine might not happen which range from institutional change to human behavioral psychology. Some of the most important can be grouped into five categories: technical feasibility, priority, human nature, timing, and criticisms of participatory preventive health models. First and foremost is technical feasibility in the sense that health research generally, whether institutional or participatory efforts, has not been effective at finding solutions to the most complex health challenges (a recent high-profile example is cancer immunotherapy). An earlier example discussed was that of genomics, that SNP analysis so far has accounted for less than 5% of disease causality [24], and epigenetics and structural variation may explain more, but are not likely to offer a short ride to translational therapies. Many other therapies and research have not led directly to cures, but rather to a slowly accruing understanding of the complexities of biology. There is little idea in some cases such as complex common disease how comprehensively effective interventions may be developed. The detailed nature of the underlying biology and its adaptive dynamical behavior—biology itself—is one of the biggest barriers to progress. There is nothing inherent in preventive medicine and participatory health to indicate that these models would be any more be successful than traditional models. However, it also does not seem that nothing would be learned from having orders-of-magnitude more data and participants at every stage of a pathology’s development, and that these new resources could eventually facilitate the resolution of challenging health problems. 

Second, preventive medicine may not be seen as a priority, or as a way to resolve other seemingly more pressing challenges such as the high cost of health care, aging populations, and systemic health issues where answers do not seem to be forthcoming. The tremendous growth in lifestyle diseases like the worldwide ‘diabesity’ epidemic may prove to be too intractable for preventive medicine to have an impact. Third, preventive medicine may not work due to human nature. Since we are human, behavior change is extremely difficult, and we experience the world more through narratives than rationality. We think in certain ways where it is hard to get anyone focused on a longer-term problem, or on statistical or probabilistic data [64]. We need social narratives, stories, and poetry to promulgate health maintenance and behavioral change, likely together with financial incentives. Programs like Safeway’s paying employees to maintain or improve health have been demonstrated to be effective [65], but have not become widespread. The effect of other initiatives remains to be seen such as the New York soda ban (on soft drinks more than 16 ounces in size) [66], and the impact of a recently-approved anti-obesity drug [67]. 

Fourth, there could be a timing issue with preventive medicine. It might be too early, both in the curve of technology development (many of the relevant technologies are still expensive and under development (e.g., whole human genome sequencing, and microbiome, transcriptome, and epigenetic profiling)), and since science has not yet translated these emerging data streams into deployable preventive medicine interventions. Additionally, another dimension, mindset and cultural milieu is similarly not yet focused on wellness maintenance and preventive medicine as a core goal that may be achieved with relatively simple programs. Finally, criticisms and identifications of the problematic aspects of participatory preventive health models have arisen including the conduct, quality, necessity, and oversight of these efforts [21].

## 4. Conclusion

It cannot be underscored how fundamentally the health landscape may need to change to accommodate Health 2050: Preventive Medicine. Numerous scientific, information technology, mindset, and institutional changes are needed. The attempted descriptions here of the wider concept of health and health care (Figure 1), and the focus on the 80% of the life cycle of a condition when it is still pre-clinical and perhaps avoidable (Table 1) comprise a nascent sketch of how to begin. What is encouraging is that growth is already evident in different areas of the preventive medicine ecosystem. One example of the expanded concept of health in action is the distinction businesses are making by defining themselves as being in either the wellness or medical sector. Consumers too are more active, and engagement in participatory health efforts could continue to grow as devices, applications, and other technologies make data collection, health-monitoring, and behavior change easy, inexpensive, and unobtrusive. The growth of big health data streams generated both by institutions and individuals could facilitate the greater use of typing groups akin to blood type groups, and predictive profiling for early warnings of out-of-baseline variation. Crowdsourced health studies conducted individually and in groups are emerging as an important complement to traditional clinical trials and other established forms of health knowledge generation. Qualitative changes in mindset may be a forerunner to institutional recasting as individuals increasingly take the responsibility to self-manage health in a more empowered proactive manner. The individual has become the central focal point in health, which is now seen as a systemic complexity of wellness and prevention, as opposed to an isolated condition or pathology. Not only is scientific advance critical, but also the philosophical and cultural context for moving away from the fix-it-with-a-pill mentality to the empowered role of the biocitizen in achieving the personalized preventive medicine of the future. 
